# Row for Your Life: A Century of Mortality Follow-Up of French Olympic Rowers

**DOI:** 10.1371/journal.pone.0113362

**Published:** 2014-11-17

**Authors:** Juliana Antero-Jacquemin, François Deni Desgorces, Frédéric Dor, Adrien Sedeaud, Amal Haïda, Philippe LeVan, Jean-François Toussaint

**Affiliations:** 1 Institut de Recherche bioMédicale et d'Epidemiologie du Sport (IRMES), Institut National du Sport de l'Expertise et de la Performance (INSEP), Paris, France; 2 Université Paris Descartes, EA 7329, Sorbonne Paris Cité, Paris, France; 3 Comité National Olympique et Sportif Français (CNOSF), Medical Commission, Paris, France; 4 Institut National du Sport de l'Expertise et de la Performance (INSEP), Medical Department, Paris, France; 5 Centre d'Investigations en Médecine du Sport, Hôpital Hôtel-Dieu, Assistance Publique-Hôpitaux de Paris, Paris, France; Universidad Europea de Madrid, Spain

## Abstract

**Background/Aim:**

Strenuous endurance training required to participate in the highest sports level has been associated with deleterious effects on elite athletes' health and cardiac abnormalities. We aimed to describe overall mortality and main causes of deaths of male French rowers participating in at least one Olympic Game (OG) from 1912 to 2012 in comparison with the French general population.

**Methods:**

Identity information and vital status of French Olympic rowers were validated by National sources from 1912 to 2013 (study's endpoint) among 203 rowers; 52 out of 255 (20.3%) were excluded because their vital statuses could not be confirmed. Main causes of deaths were obtained from the National registry from 1968 up to 2012. Overall and disease-specific mortalities were calculated through standardised mortality ratios (SMRs) with its 95% confidence intervals (CIs). The overall mortality was calculated for the whole rowers' cohort (PT) and for two periods apart: (P1) including rowers from 1912 to 1936 OG, a cohort in which all rowers have deceased and (P2) considering rowers from 1948 to 2012 OG.

**Results:**

Among the 203 rowers analysed, 46 died before the study's endpoint, mainly from neoplasms (33%), cardiovascular diseases (21%) and external causes (18%). PT demonstrates a significant 42% lower overall mortality (SMR: 0.58, 95% CI: 0.43–0.78, p<0.001), P1 a 37% reduction (SMR: 0.63, 95% CI: 0.43–0.89, p = 0.009) and P2 a 60% reduction (SMR: 0.40, 95% CI: 0.23–0.65, p<0.001) compared with their compatriots. Mortality due to cardiovascular diseases is significantly reduced (SMR: 0.41, 95% CI: 0.16–0.84, p = 0.01) among rowers.

**Conclusions:**

French Olympic rowers benefit of lower overall mortality compared with the French general population. Among rowers' main causes of death, cardiovascular diseases are reduced in relation to their compatriots. Analytical studies with larger samples are needed to understand the reasons for such reductions.

## Introduction

Rowing has been reported as a safe contactless sport with low injury rate, providing health benefits to its practitioners [1], [2]. Although high occurrence of cardiac diseases, and alterations on cardiovascular morphology has been previously reported in university rowers [3], [4], epidemiological studies have described higher life expectancy in Harvard and Yale university rowers as in Oxford-Cambridge boat racers compared with their non-athletic referents [5], [6].

If regular physical activity has been demonstrated to provide large health benefits [7]–[9] studies have reported that strenuous exercises and training of elite athletes could be associated with deleterious effects [10]: cardiac abnormalities [11], [12] more likely triggering a sudden death during high demanding effort [13], [14], with higher incidence rate among men varying according sports practice [15] and deregulated inflammatory responses [16]. In addition, training cessation after international career has showed to be deleterious in retired athletes [17]–[20]. Conversely, a recent meta-analysis of epidemiological studies has concluded that top-level athletes live longer than the general population [21].

Olympic Games (OG) are the highest competition level for rowing athletes; thus Olympians are exposed to the most extraneous training program and performance exigencies. This study aims to assess the overall mortality and main causes of deaths of male French Olympic rowers competing from 1912 up to 2012 compared with their referents in the French population.

## Methods

### Design and participants

Retrospective cohort study of French Olympic rowers. The cohort was composed of all male rowers who represented France in at least one OG, from 1912 up to 2012, and had their vital status validated by the National registry of Identification of Physical Persons (RNIPP). Coxswains were not accounted as rowers.

**Figure 1:**
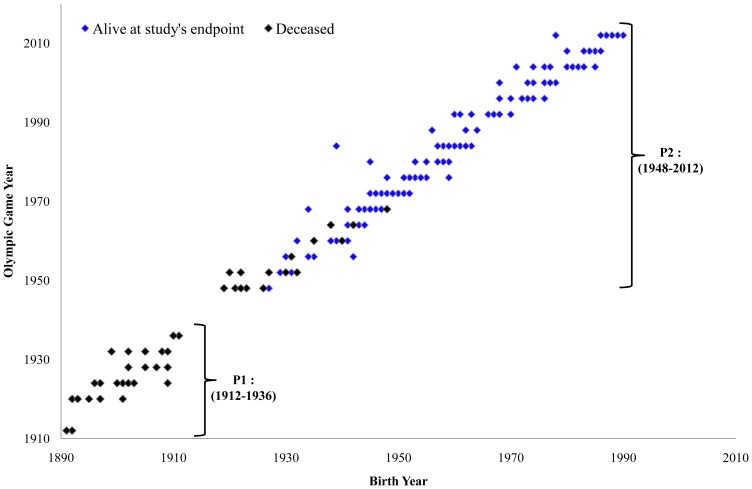
French Olympic rowers by Olympic Games and birth year. Black diamond represents deceased subjects and blue diamond represents the subjects alive at the study's endpoint. The two periods studied apart are schematically indicated.

Three periods were taken into account to analyse rowers' overall mortality. The first (P1) included only rowers that participated in the OG from 1912 up to 1936. This period was chosen for a separated analysis because all rowers, who participated in those games, had already deceased, allowing for a complete follow-up until the entire cohort's extinction. The second period (P2) is an uninterrupted period of OG after World War II, from 1948 to 2012. Finally, we considered the total period (PT), analysing the totality of rowers participating in the OG from 1912 up to 2012 (Figure 1). The discontinuity at the rowers' entry in the cohort between 1912–1920 and 1936–1948 is related to the Games interruption due both World Wars.

### Data collection

Data concerning rowers' biography came from historians' sources as previously reported [22]. These records were confirmed by data of the International Olympic Committee (olympic.org) and the French Federation of Rowing (http://www.avironfrance.asso.fr/). Rowers' identity information and vital statuses were collected since their first Olympic participation up to January 1st 2013 (study's endpoint) from the RNIPP via INSEE (National Institute of Statistics and Economical Studies), which registers vital status of all French people since 1891. Hence, rowers born before 1891 were not included in the study.

The causes of death of each deceased subject were obtained from the CépiDc (Epidemiologic Centre on Medical Causes of Death). Currently, causes of deaths occurring on French territory are available via National registry from 1968 up to January 1^st^, 2012. Thus, only deaths occurring during this period were submitted to investigation of its causes.

This study was approved by the Advisory Committee on Information Processing in Research in the Field of Health (CCTIRS) and the French National Commission for Data Protection and Liberties (CNIL).

### Statistical Analysis

We compared the mortality of French rowers with the French general population by calculating overall and disease-specific Standard Mortality Ratio (SMR) and its 95% confidence intervals (CI). SMR is the ratio between the number of deaths observed in the rowers' cohort and the number of expected deaths if the athletes had the same death rates of the French population with age and period adjustment [23]. The source for general population death rates was the French life tables available at the Human Mortality Database (http://www.mortality.org/) via CépiDc.

The number of expected deaths was therefore calculated by multiplying male French death rates by the rowers' person-years at the corresponding age and period. Person-years were computed so that each rower contributes for one record of each year during his follow-up.

The rowers' follow-up starts with their date of first OG participation and finishes with their date of death (if concerned) or with the study's endpoint. The rowers' entries in the cohort are represented in Figure 1.

The observed and expected survival estimations were illustrated by Kaplan-Meier curves [24] and were compared with use of the log-rank test.

For the disease-specific mortality we compared each cause of death found among rowers with the specific rate in the general population by calculating SMR's for the available identification period, 1968 to 2012. Causes of death were classified according to the International Classification of Diseases - ICD (8th revision before 1978, 9th revision between 1979 and 1999, 10th Revision after 2000).

The 95% confidence intervals were calculated using the exact method [25]. R software v2.14.0 was used for the analysis.

## Results

### Overall mortality

A total of 255 male rowers represented France in the Olympics from 1912 up to 2012. All rowers verified by the RNIPP had their vital status validated, except for fifty-two (20.3%) who were not included in the study since they were born prior to 1891 (n = 10) or their date or place of birth were not properly validated (n = 42). Therefore, the analyses were performed on 203 French rowers.

Among rowers' cohort, 46 deaths occurred before the study's endpoint; 30 within P1 (Table 1). The age of death ranged from 35 to 100 years old. The mean age of death was 72.5 (±15.6) in P1 and 74.0 (±9.1) in P2. The PT overall mortality is significantly reduced by 42% compared with the French general population. Significant overall mortality reduction is also observed among rowers for both cohort periods considered apart (Table 1).

**Table 1:** French Olympic rowers' cohort description and their overall mortality compared with the French general population.

Cohort	PT (n = 203)	P1 (n = 30)	P2 (n = 173)
Cohort's entry period	1912–2012	1912–1936	1948–2012
Nb. of person-years	6724.2	1528.4	5195.8
Mean age at cohort's entry in years (SD)	24.1 (±3.6)	24.1 (±3.7)	24.1 (±3.6)
Mean follow-up time in years (SD)	33.8 (±18.5)	48.4 (±15.7)	31.0 (±17.7)
SMR (95% CI)*	0.58 (0.43–0.78)	0.63 (0.43–0.89)	0.40 (0.23–0.65)
p-value	<0.001	0.009	<0.001

*SMR lower than 1.0, indicates a reduced mortality of rowers compared with the reference population.

**Figure 2:**
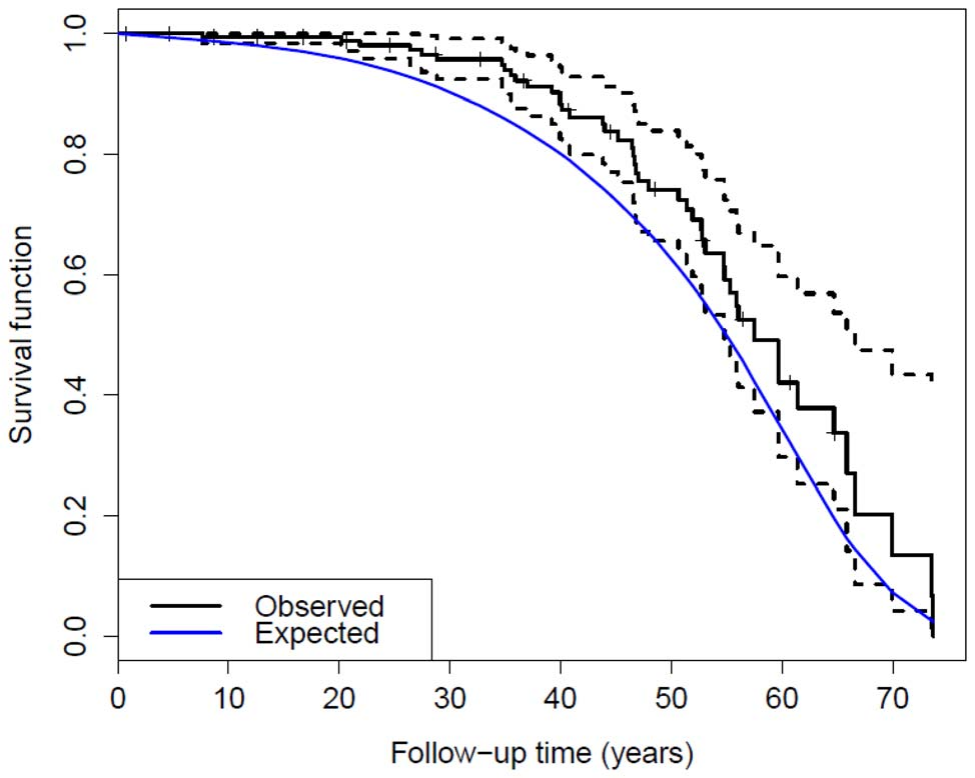
French Olympic rowers' observed and expected survival curves. Rowers' observed survival is represented by the solid black curve and its dashed confidence interval. The blue curve refers to the expected survival derived from the matched general French population.

The rowers observed and expected survival curves are illustrated in Figure 2. The rowers observed curve is significantly different from the expected curve derived from the matched general French population (p = 0.008). The observed rowers' curve diverges and shifts to the right early in the follow-up period. The gap widens until 45 years of follow-up, and narrows afterwards until the end of follow-up. With increasing time, progressively fewer rowers contribute to the survival estimate.

### Main causes of death

Among the 46 deaths observed, 36 occurred during the available period of death's cause identification (1968–2012). From those, 3 died abroad and their cause of death were not available. Therefore, 33 rowers had their underlying cause of death identified. The main causes of death (Table 2) observed were neoplasms, n = 11(33%), followed by cardiovascular diseases n = 7 (21%) and external causes n = 6 (18%). The ICD chapter of external cause comprises deaths related to accidents, homicides, suicides and falls. The remaining causes observed (nervous system diseases, genitourinary diseases, respiratory diseases, skin diseases and ill-defined conditions) together account for 27% of all causes of death identified. Concerning the three main causes observed among French Olympic rowers, cardiovascular diseases are 59% significantly lower than found in the general population. Neoplasms and external causes are not significantly different from the general population (Table 2).

**Table 2:** French Olympic rowers' main disease-specific mortality compared with the French general population.

Main causes of death (n)	Observed/Expected deaths	SMR (95% CI)	p-value
Neoplasms (11)	11/18.4	0.59 (0.29–1.07)	0.09
Cardiovascular diseases (7)	7/17.0	0.41 (0.16–0.84)	0.01
External causes (6)	6/5.7	1.0 (0.38–2.29)	0.99

## Discussion

To our knowledge the present study is the first to describe a survival advantage of elite rowers compared with their referents in the general population. The follow-up over the last century shows a 42% reduction of the overall mortality of French Olympic rowers. The early cohort of rowers (P1) followed until its complete extinction as well rowers competing post Wars up to 2012 (P2) showed a consistent mortality reduction.

The difference between observed and expected survival curves since the beginning of the follow-up illustrates the rowers' survival advantage over the general population. This advantage, visible since the beginning of the follow-up, increases with time and rowers deviate most from the expected when they are 50 to 70 years old. At older ages the rowers' survival approaches the curve of their compatriots.

The increased selectivity of the Olympics in the modern era may require increased training intensity and may further develop the use of enhancing performance techniques, with potential adverse effects on athletes' health [26]. The consequence of such period changes on elite rowers' mortality will only be fully acknowledged after the entire extinction of the current young rowers, as it will allow a complete retrospective analysis. Up to now, our results suggest that recent rowers' longevity (P2) follows the survival advantage trend observed among P1 rowers; the mortality difference with the general population even seems to widen. However our results should be cautiously interpreted as we compare healthy individuals at cohort's entry with the general population, including ill and handicapped individuals. This “healthy worker effect” [27] probably overestimates the mortality reduction found among rowers.

French rowers' survival advantage appears associated with a reduced mortality from cardiovascular diseases. The benefits of leisure endurance sports on the cardiovascular health are well known in the general population [28], [29] but some studies have underlined possible risks to the cardiovascular system due to excess training [13], [30], [31]. The preparation to OG includes important training volumes with rowers reaching more than 1000 h of training per year [32]. Such training loads undertaken during years of practicing produces major adaptations of the athletes' cardiovascular system [4], [33] a phenomenon probably enhanced by a previously selection process [34], [35]. Among French Olympic rowers, rather than a risk, the cardiovascular adaptation to training requirements to access the OG seems to be protective in light of the 59% mortality reduction found among them. Previous studies assessing cardiovascular diseases of endurance elite athletes as defined by the ICD show: a 51% reduction among Finish long distance running and cross country skiing [36] and a 37% reduction among French cyclists [37]. Finally, our findings among Olympic rowers are also in line with longevity studies assessing rowers from lower competitive level [5], [6].

Similar reduced overall mortality have been reported among other elite athletes cohorts [21], [36], [38], [39], including recent findings considering endurance athletes [40]. A study of French cyclists in the *Tour de France* with comparable methods - although the number of subjects and follow-up period are different - has shown a 41% reduction of cyclists overall mortality [37]. Although road cycling risks are greater than rowing [2], French rowers and cyclists presents similar mortality advantage over their compatriots. Yet, the lower injury incidence among rowers might be behind the contrast with American football players' mortality [41]. Despite an overall mortality reduction of 47% the players presented an increased mortality due to neurodegenerative diseases claimed to be related with concussive blows to the head.

We limited our cohort to Olympic athletes in order to focus on the highest sports level for which health consequences of intense training remains debatable [10]. Nevertheless, our results are based on a small absolute number of subjects, especially those concerning causes of death; hence they should be interpreted with caution. In addition, we are not able to determine how the excluded athletes might alter the magnitude of our findings. Finally, except for age and period, we could not adjust for potential confounding factors. Our methods advantage, however, relies on an extensive follow-up of French Olympic rowers followed during a century of OG, allowing for a complete follow-up of the rowers competing in the first Games. In addition, the vital status and causes of deaths assessed and validated by National sources reduces uncertainty.

Further investigations based on larger cohorts and in a large variety of countries are now needed to better apprehend elite athletes' reduced mortality. As it is out of an observational study's scope to determine causality, analytical studies are necessary to explain elite athletes' survival advantage. The assessment of lifestyle or physical activity post-career of elite athletes may provide better understanding of their mortality determinants.

## Conclusions

French elite rowers followed-up during a century of Olympic Games benefit of lower overall mortality compared with the French general population. The cohort composed only of deceased rowers from the early Olympic Games analyzed apart and followed until its complete extinction shows also an important mortality reduction. Among elite rowers' main causes of death, cardiovascular diseases are reduced in relation to their compatriots. Analytical studies with larger samples controlling for confounders such as elite athletes' training-load and lifestyle after career are needed to understand the reasons behind elite athletes' survival advantage.
